# Leukaemia*Paper read before the Long Island Veterinary Society Jan. 21, 1891.

**Published:** 1891-03

**Authors:** George G. Vandeveer


### LEUKAEMIA.*

BY DR. GEORGE G. VANDEVEER.

The earliest investigations as to the nature of this disease are those of Bennett and Virchow which were published in the Autumn of 1845. Bennett supposed that the altered condition of the blood was due to the presence of pus and it was not until six years later, 1851, that he abandoned his theory and gave the name leucocythaemia to the disease.

Virchow's researches led him from the first to assign the cause of this white blood to leucocytes. Two years later he published an article in which he considered the conditions under which there might be an increase of the white cells, and their relation to the spleen, and also proposed the name leukaemia.

In 1853 Virchow separated two forms, splenic and lymphatic, and in 1869 Neumann discovered a myelogenous form.

Leukaemia is characterized by a great and persistent increase in the white blood corpuscles, together with a simultaneous decrease in the red cells, but it is unknown whether this relation is due to an arrest of the transformation of white into red cells, or to an increased supply of white cells, or whether both of these causes act together.

Little is known as to the causes of the disease, but all the factors which tend to produce primary diseases in spleen, lymphatic glands and bone-marrow should be considered. As to climate, more cases have been reported in temporate regions than in the tropics.

It attacks all ages, but males are more prone to its ravages than females in the proportion of at least 3–1. It is more common among the poorly housed and fed, than among those who enjoy better sanitary surroundings.

Previously existing diseases may have an effect in producing the disorders prominent among which is mentioned malaria, while pre-existing haemorrhages, and injuries from blows and strains are frequent in the histories of cases.

There is extreme wasting often, oedema is common, and ascites may be present.

The full amount of blood in the heart and blood vessels

*Paper read before the Long Island Veterinary Society Jan. 21, 1891.

usually in the form of large clots is a noteworthy feature, and the collections of white cells densely infiltrating these clots, present a pus-like appearance.

In the majority of cases the spleen is hypertrophied ; the splenic tumor is always of considerable size, and generally retains its normal form, developing proportionally in all its dimensions. It is usually bluish-red in color, and may be united to the abdominal-wall diaphragm or stomach by strong adhesions. The capsule is thickened and the vessels enlarged. The organ is hard and firm and cuts with resistance, and grayish-white granular tumors may occur throughout its whole extent, either scattered about or arranged in rows. In the early stages there is swelling of the pulp, and increase in the cell elements without the firmness and hardness of the fully developed leukaemic organ, and at this period rupture may occur.

Uncomplicated cases of the lymphatic form are rare, usually the lymphatics enlarge with the spleen, and in the majority of instances the hypertrophy is not extensive. The process seems, just as in the spleen, to begin with a greater flow of blood, and an increased vascularization, under the influence of which the multiplication of glandular elements takes place. The groups of axillary, mesenteric and inguinal glands are the most frequently affected, they are moderately soft, movable and isolated ; in chronic cases they may become very indurated.

The bone-marrow is usually the seat of important changes ; both in the central cavity of the long bones and in the cancellated structure of the ribs, the sternum and the vertebræ, the marrow has the same greenish-yellow, purulent color, and the same consistency as mucus pus. Under the microscope it may be seen that cellular elements of the same nature as those occurring in leukaemic blood, form the principal constituents.

The liver is very commonly enlarged, it is pale, smoothe and retains its shape. The substance is usually firm, of a grayish-brown color or may be marbled. Two chief changes have been met with—a diffuse leukaemic infiltration, and numerous small leukaemic tumors.

The kidneys are usually pale, and often enlarged, the capillaries may be distended with leucocytes, and leukaemic tumors may be found generally in the cortex.

The respiratory system is not often the seat of important lesions. Lymphoid growths have been found in the mucus membrane of the trachea and bronchi, and occasionally in the lungs, where they may closely resemble tubercles, but differ from them in having no tendency to caseate or soften.

In the digestive system, the stomach rarely presents any change other than catarrhal ; the intestines have in many cases been the seat of tumors which have originated in the solitary and agumated glands. In a few cases the bowel lesions have been so pronounced that the term intestinal leukaemia seemed justifiable.

The changes in the blood are so characteristic that they form the most prominent mark of the disease ; the lighter color is more and more marked as the disease progresses ; the increase of leucocytes is continued and progressive, leading in its regular progress to the death of the patient. The proportion of colorless corpuscles finally reached is very great as high as 1–3 or 1–2. The red corpuscles are not only relatively, but absolutely diminished. The specific gravity is lowered ; the water and febrine are increased, and iron considerably diminished.

The most prominent symptoms are weakness, exhaustion, difficulty in breathing, paleness, and emaciation and profuse sweating. Among the most striking symptoms of splenic leukaemia, are to be reckoned haemorrhages. The intestinal evacuations are interfered with, at first constipation alternates with diarrhœa, while later the diarrhœa predominates, becomes copious and frequent, and sometimes bloody. The urine excretion is in most cases normal in quantity, but toward the end always diminished.

In most cases a considerable disturbance in the temperature is manifested, in the early stages there is only slight variation, but when the disease is well advanced there is always fever of the remittent or of the continuous type.

The appetite, in most cases normal, is rarely diminished, but is sometimes much increased.

The complications may be summed up as serous or sanguino-serous exudations into the cranial, pleural, and abdominal cavities, oedema and congestion of the lungs, pleuritic and peritoneal inflammations and adhesions, and thrombi from the plugging up of the vessels by leucocytes.

The course of the disease is slow and chronic. In exceptional instances, usually in young subjects, it runs a rapid course, but acute leukaemia is rare.

Death takes place usually by asthenia—a gradually progressive weakness, and finally failure of the heart. Diarrhœa and haemorrhages hasten the result. Pyaemia and rupture of the spleen are mentioned as causes of death in some cases.

The positive diagnosis depends upon the determination of a great and persistent increase in the white blood corpuscles.

The prognosis is unfavorable in the highest degree ; when firmly established, the spleen and glands are enlarged, the blood condition is marked, and haemorrhages and dropsics are present, death is the only termination to be expected.

